# Supplementary material for: Scalable production of ultrafine polyaniline fibres for tactile organic electrochemical transistors
Source: Nat Commun. 2022 Apr 19;13:2101. doi: 10.1038/s41467-022-29773-9 (PMC9018749; doi:10.1038/s41467-022-29773-9)
Supplement: Supplementary file 1 — Supplementary Information [file 41467_2022_29773_MOESM1_ESM.pdf]

Supplementary information for

Scalable production of ultrafine polyaniline fibres for tactile organic electrochemical transistors

Bo Fang[1,2,&,*], Jianmin Yan[1,3,&], Dan Chang[4], Jinli Piao[1,2], Kit Ming Ma[1,2], Qiao Gu[5,6], Ping Gao[5,6], Yang Chai[1,3]* Xiaoming Tao[1,2]*

[1]Research Institute for Intelligent Wearable Systems, The Hong Kong Polytechnic University, Hong Kong, 999077 China.

[2]Institute of Textiles and Clothing, The Hong Kong Polytechnic University, Hong Kong, 999077 China.

[3]Department of Applied Physics, The Hong Kong Polytechnic University, Hong Kong, 999077 China.

[4]Department of Polymer Science and Engineering, Zhejiang University, Hangzhou, 310027 China.

[5]Department of Chemical and Biological Engineering, The Hong Kong University of Science and Technology, Hong Kong, 999077 China.

[6]Advanced Materials Thrust, The Hong Kong University of Science and Technology (Guangzhou), Guangzhou, 510000 China.

[&]These authors contributed equally.

***Email: bofang@polyu.edu.hk; ychai@polyu.edu.hk; xiao-ming.tao@polyu.edu.hk**

Inventory of Supplementary information

Supplementary Fig. 1: Photograph of continuous collecting of ultrafine polyaniline fibres (UFPFs).

Supplementary Fig. 2: The surface and cross-section of UFPFs at different magnitudes.

Supplementary Fig. 3: X-ray diffraction spectra of UFPFs collected from DMF bath and rough PAni fibres collected from acetone bath.

Supplementary Fig. 4: The relationship between length and resistance of PAni fibres.

Supplementary Fig. 5: Mechanics simulation.

Supplementary Fig. 6: Volumetric capacitance, power and energy density of UFPFs.

Supplementary Fig. 7: The identification of water window in micro capacitor.

Supplementary Fig. 8: The voltage-current relationship at a scan rate of 100 mV s^{-1}.

Supplementary Tab. 1: The electrical properties in fibre-based OECTs.

Supplementary Fig. 9: The device measuring the friction response of OECT.

Supplementary Reference

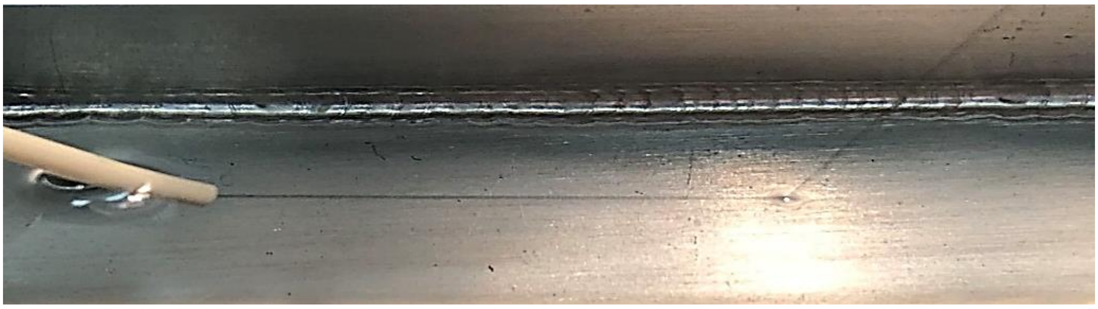

Supplementary Fig. 1 | Photograph of continuous collecting of ultrafine polyaniline fibres (UFPFs) in dimethyl formamide (DMF) bath. A clear slenderization occurs along the fibre length.

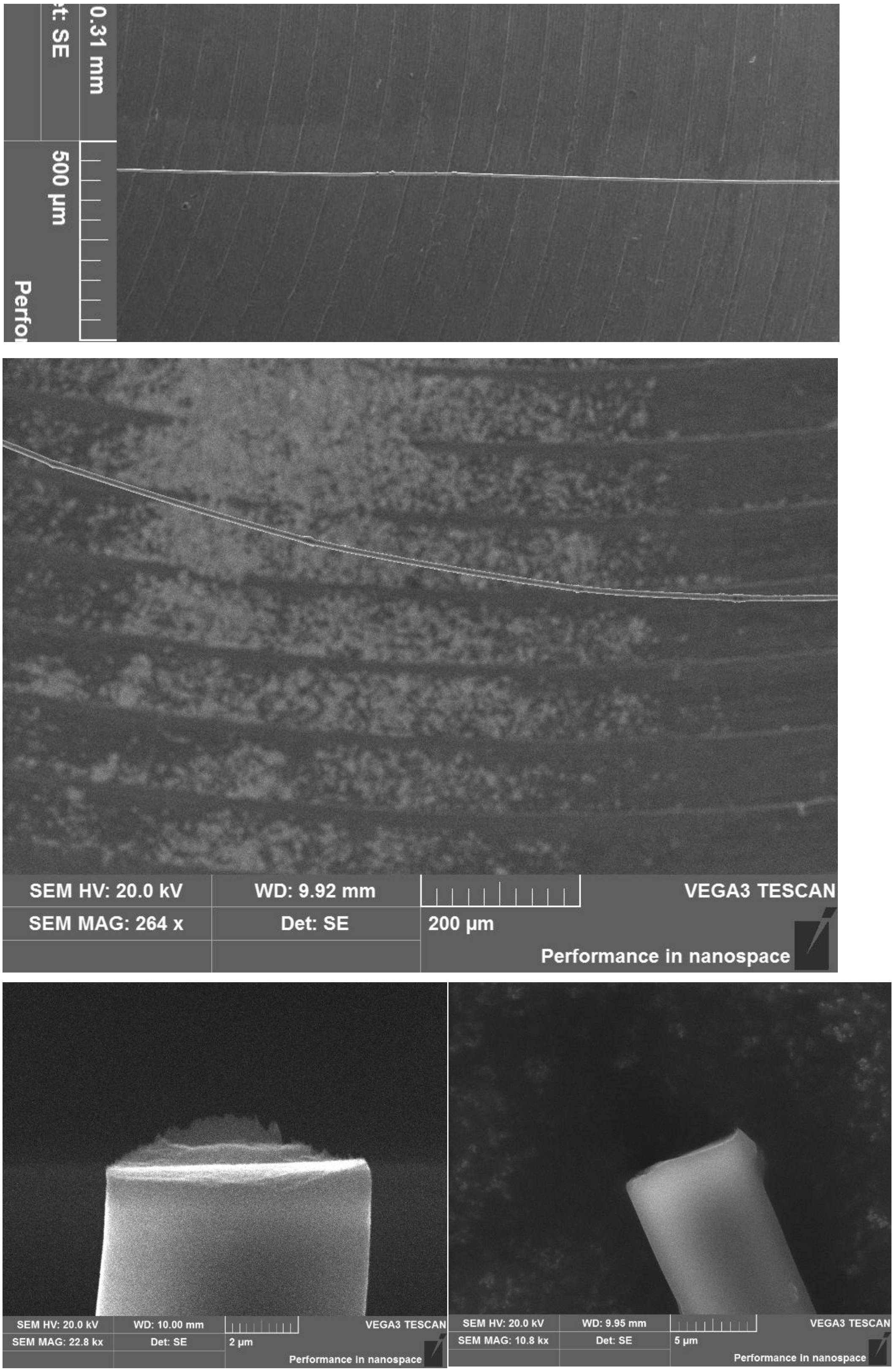

Supplementary Fig. 2 | SEM images showing the surface and cross-section of UFPFs at different magnitudes. The smooth surface along the fiber length and the uniform section identifies the favorable structural uniformity of UFPFs.

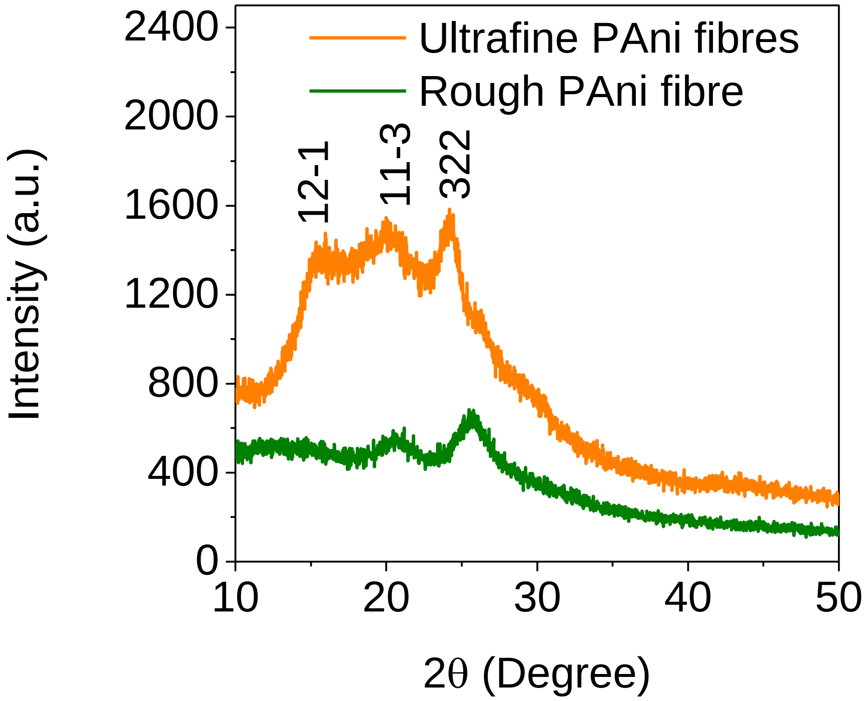

Supplementary Fig. 3 | X-ray diffraction spectra of ultrafine PAni fibres collected from DMF bath (orange curve) and rough PAni fibres collected from acetone bath (olive curve). Comparing to the rough fibres, the ultrafine PAni fibres exhibit rich crystalline peaks along the 12-1, 11-3 and 322 panels, suggesting the highly crystallized microstructures of UFPFs.

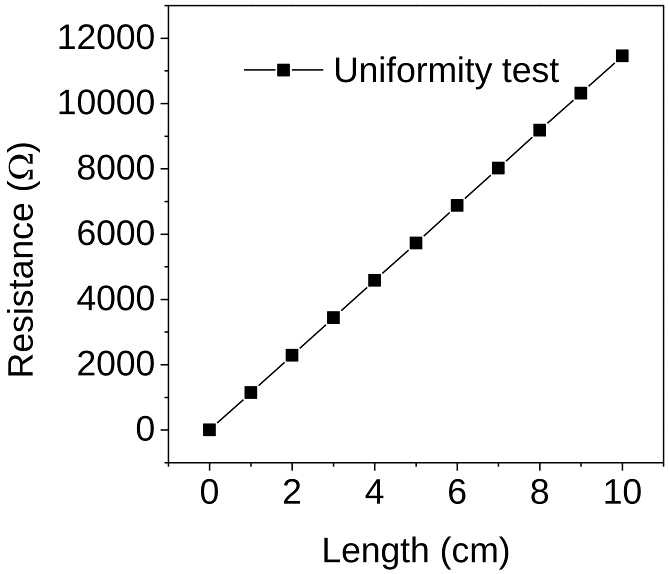

Supplementary Fig. 4 | The relationship between length and resistance of PAni fibres with increasing lengths from 1 cm to 10 cm. The linear relationship between length and resistance suggests a uniform charge distribution along the fibre length.

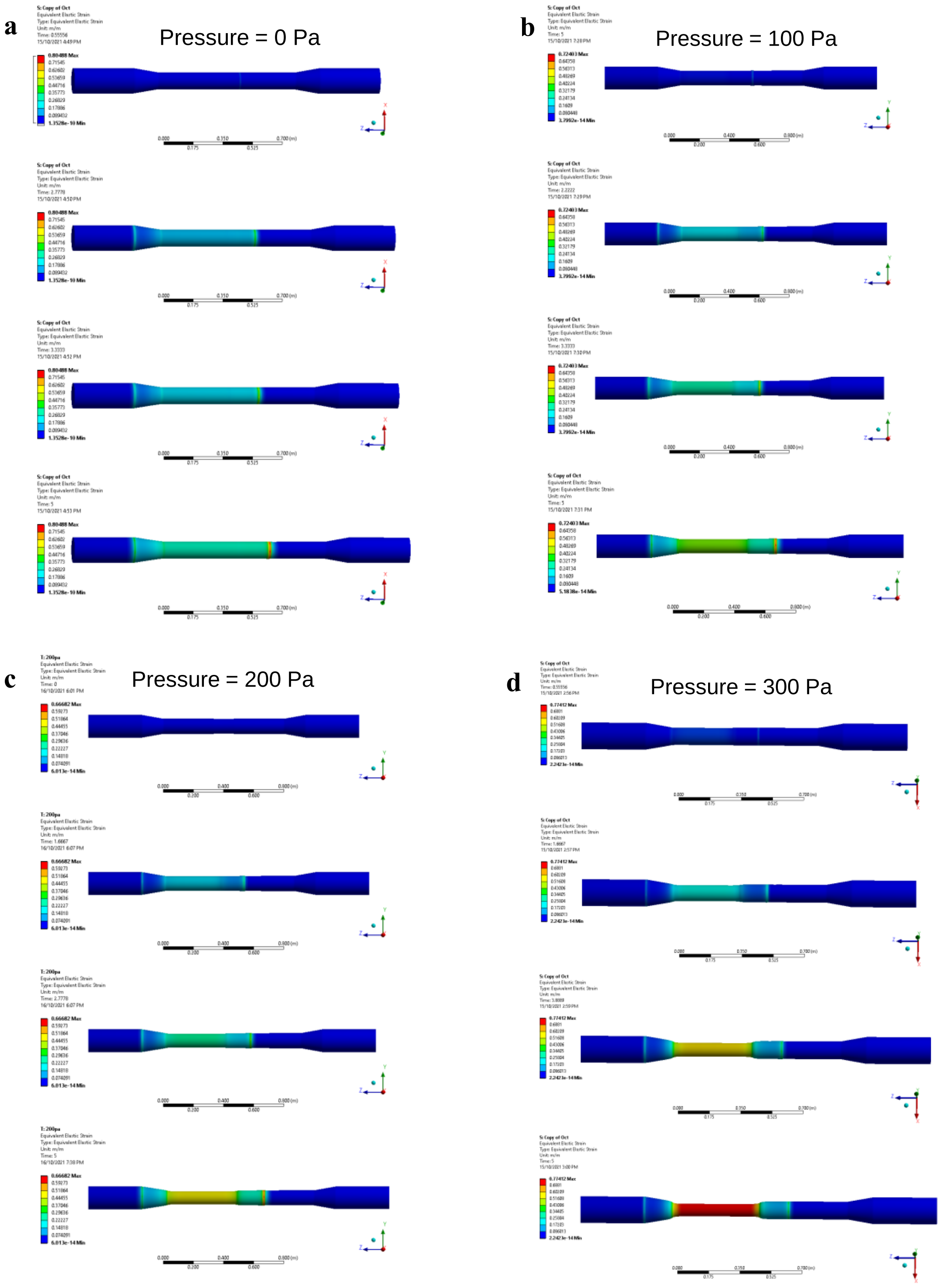

Supplementary Fig. 5 | Mechanics simulation to the progressive extension behaviors of PAni gel protofibres at different interfacial pressures increasing from 0 (a), 100 Pa (b), 200 Pa (c) to 300 Pa (d). Obviously, the necking phenomena tend to occur at the higher interfacial pressures.

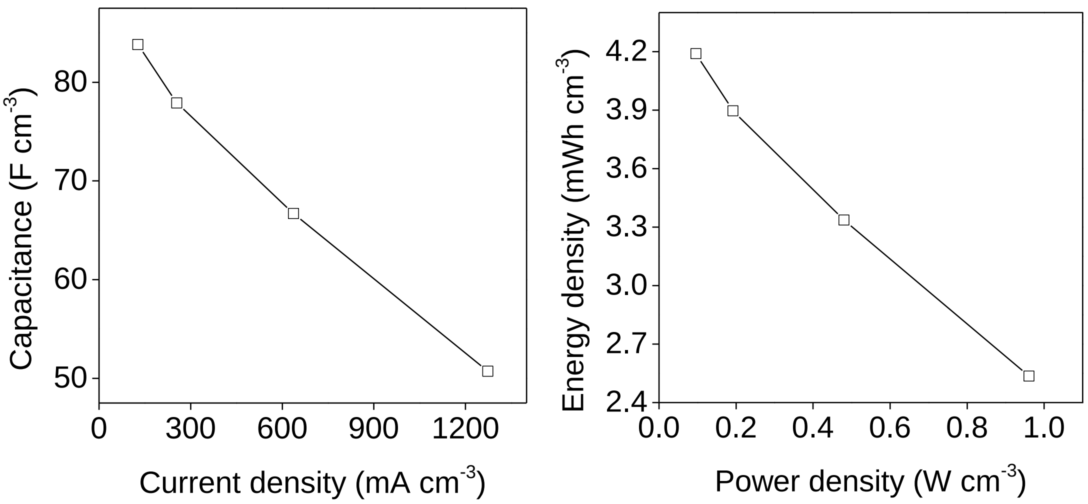

Supplementary Fig. 6 | Volumetric capacitance, power and energy density of UFPFs. At the current densities between 127.4 and 1273.8 mA cm^{-3}, the volumetric capacitance is calculated to be between 83.8 and 50.7 F cm^{-3}, the power densities between 0.1 and 0.96 W cm^{-3}, and energy densities between 2.53 and 4.19 mWh cm^{-3}.

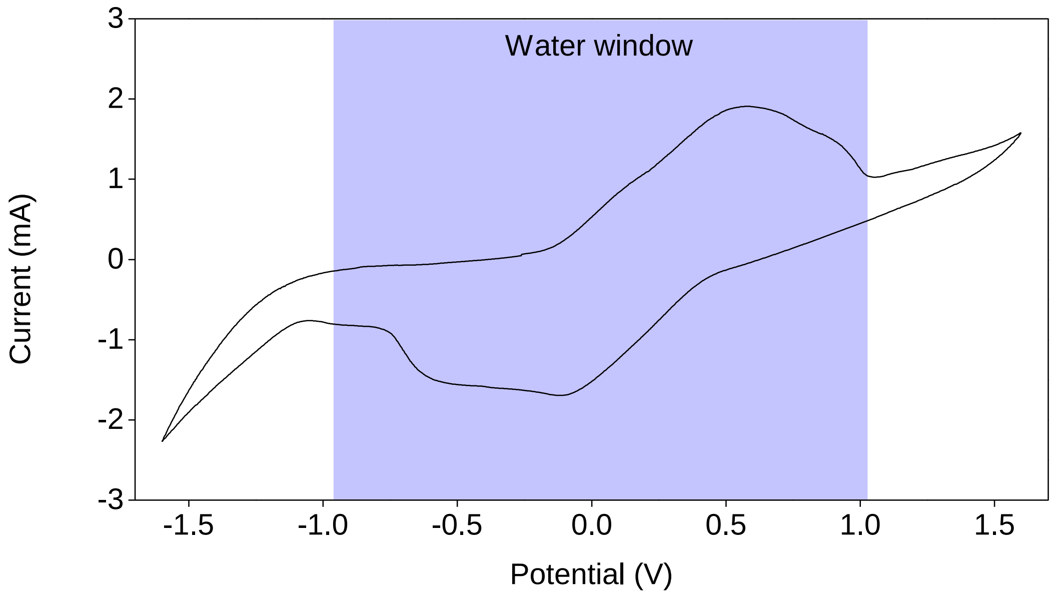

Supplementary Fig. 7 | The identification of water window in UFPF-based micro capacitor. The water oxidation and reduction potentials, indicated by the steep increase of current densities in CV curve, define the water window of micro capacitor. The water window is located between -0.9 and 1 V in UFPF-based micro capacitor.

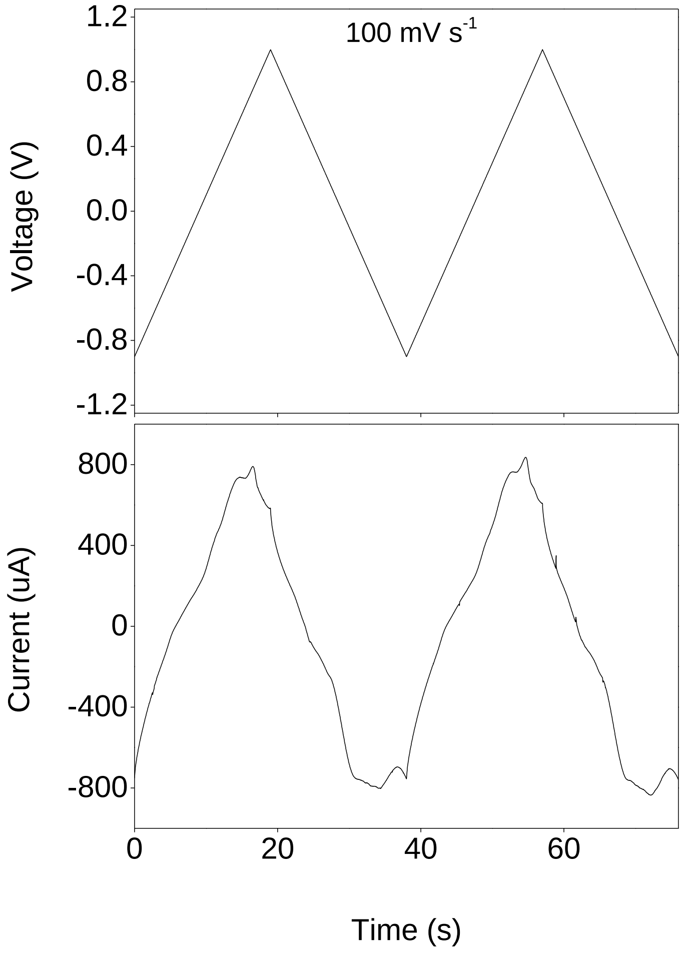

Supplementary Fig. 8 | The voltage-current relationship at a scan rate of 100 mV s^{-1}. Deriving from this curve, the area charge storage capacity and volumetric capacity is 2.015×10^4 mC cm^{-2} and 8.07×10^4 mC cm^{-3}, respectively.

Supplementary Table 1: The electrical properties in fibre-based OECTs				
Ref No.	Channel	On/Off ratio	Drive (V)	g_m (μS)
This work	PAni	10^3	0.6	60
Ref. 1[1]	PPy/PVA/PE	$2.6x10^2$	3	/
Ref. 2[2]	PPy	10^4	2	/
Ref. 3[3]	CNT	10^2	1	1350
Ref. 4[4]	PPy/Graphene	10^2	2	/
Ref. 5[5]	PEDOT/PSS	10^3	1	1000

Acronym in table: PAni (Polyaniline); PPy (Polypyrrole); PVA (Polyvinyl alcohol); PE (Polyethylene); CNT (Carbon nanotube).

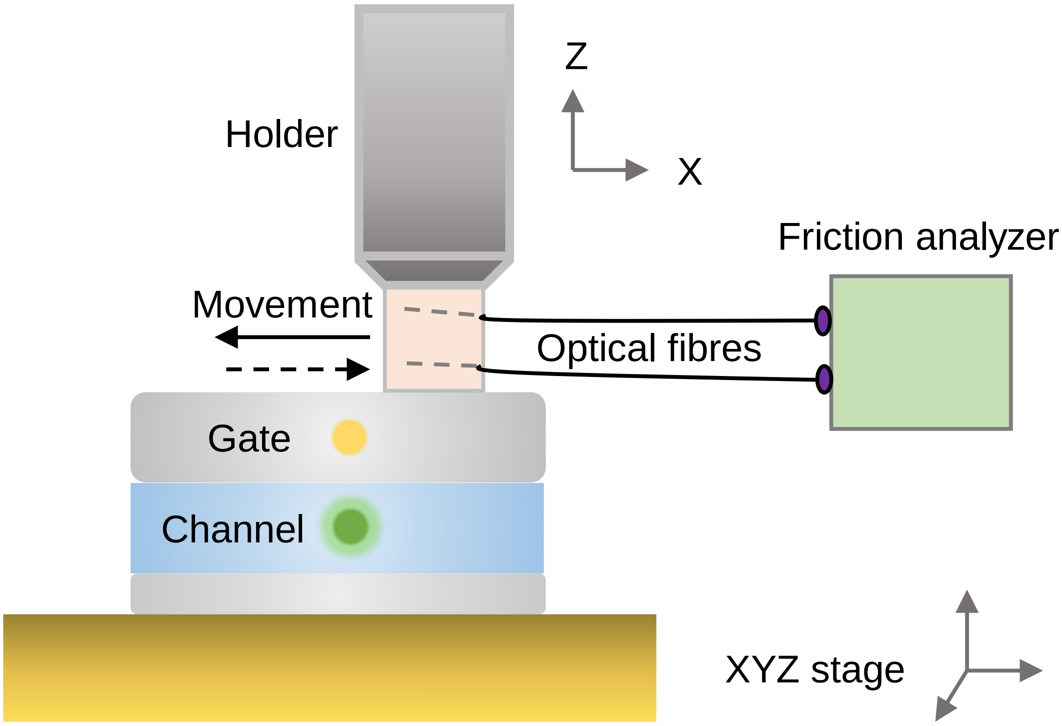

Supplementary Fig. 9 | The device measuring the friction response of OECT. The device is mainly composed of three parts: the upper holder directing the movement of friction analyzer (light red), an UFPF-based OECT right below the friction sensor connecting to a friction analyzer through optical fibres, and a XYZ-stage controlling the position of OECT. The working principle of friction analyze system referees to Zhang's work[6].

Supplementary References

1. Qing, X. et al. Wearable fiber-based organic electrochemical transistors as a platform for highly sensitive dopamine monitoring. *ACS Appl. Mater. Inter*. **11,** 13105-13113 (2019).
2. Wang, Y. et al. Ion sensors based on novel fiber organic electrochemical transistors for lead ion detection. *Anal. Bioanal. Chem.* **408,** 5779-5787 (2016).
3. Wu, X. et al. Fiber-shaped organic electrochemical transistors for biochemical detections with high sensitivity and stability. *Sci. China-Chem.* **63,** 1281-1288 (2020).
4. Wang, Y. et al. The woven fiber organic electrochemical transistors based on polypyrrole nanowires/reduced graphene oxide composites for glucose sensing.

Biosens. Bioelectron. **95,** 138-145 (2017).

5. Kim, Y. et al. Strain-engineering induced anisotropic crystallite orientation and maximized carrier mobility for high-performance microfiber-based organic bioelectronic devices. *Adv. Mater.* **33,** 2007550. (2021).
6. Zhang, Z. F., Tao, X. M., Zhang, H. P. & Zhu, B. Soft fiber optic sensors for precision measurement of shear stress and pressure. *IEEE Sens. J.* **13,** 1478-1482 (2013).
